# Supplementary material for: Expression and Characterisation of the First Snail-Derived UDP-Gal: Glycoprotein-N-acetylgalactosamine β-1,3-Galactosyltransferase (T-Synthase) from Biomphalaria glabrata
Source: Molecules. 2023 Jan 5;28(2):552. doi: 10.3390/molecules28020552 (PMC9865085; doi:10.3390/molecules28020552)
Supplement: Supplementary file 1 [file molecules-28-00552-s001.zip › molecules-2097343-supplementary.pdf]

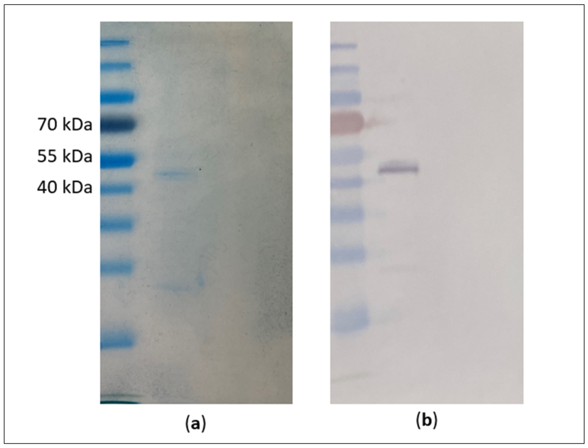

Figure S1. Expression of the recombinant protein (**a**) Coomassie staining; (**b**) Immunoblotting with Penta-His tag monoclonal antibody.

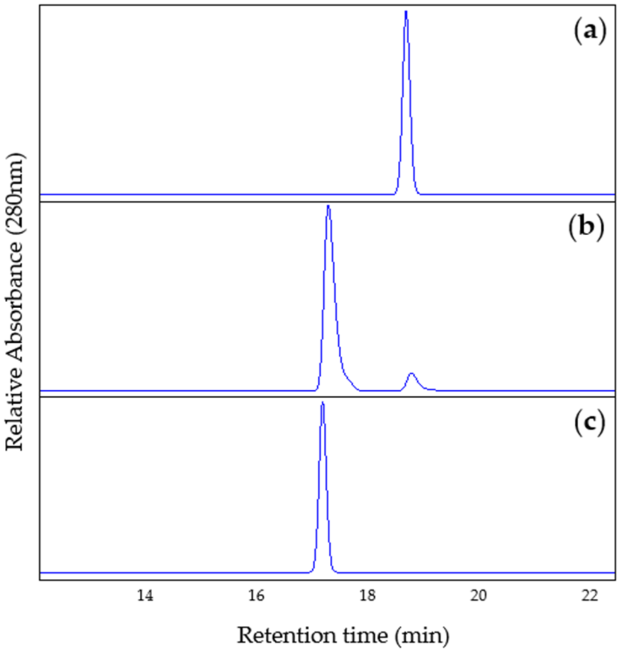

Figure S2. HPLC analysis of the transfer of Gal to pNP-α-GalNAc. (**a**) Standard pNP-α-GalNAc; (**b**) incubation for 2 hours; (**c**) Standard pNP-α-GalNAc-β-Gal.

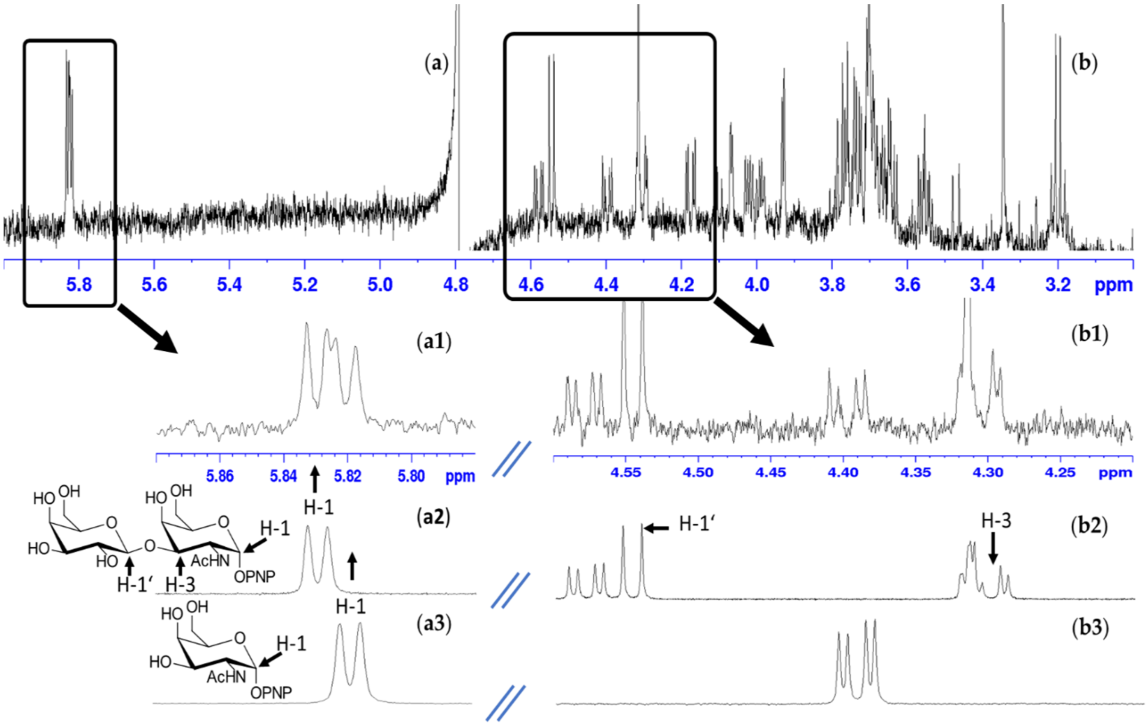

Figure S3. ^{1}H NMR of the incubation assay compared to ^{1}H NMR spectra of pNP-GalNAc and Gal-β-(1→3)GalNAc-α-pNP. (**a1**) Expanded anomeric region around 5.8 ppm shows a mixture of pNP-GalNAc (**a3**) and Gal-β-(1→3)GalNAc-α-pNP (**a2**); (**b1**) The core region between 4.6 and 4.2 ppm shows the presence of the β configured position 1 of Gal and glycosylated position 3 of pNP-GalNAc (**b2**); (**a2**) and (**b2**) show expanded ^{1}H NMR view of commercial available Gal-β-(1→3)GalNAc-α-pNP reference; (**a3**) and (**b3**) show expanded ^{1}H NMR view of commercially available pNP-GalNAc reference.
